# Case Report: A Case Study on the Neurodevelopmental Profile of a Child With Pallister–Killian Syndrome and His Unaffected Twin

**DOI:** 10.3389/fped.2022.817133

**Published:** 2022-03-15

**Authors:** Carole A. Samango-Sprouse, Mary P. Hamzik, Kenneth Rosenbaum, Kosar Khaksari, Francie Mitchell, Ritika Kommareddi, Michaela R. Brooks, Elizabeth Tipton, Teresa Sadeghin, Andrea L. Gropman

**Affiliations:** ^1^Department of Pediatrics, George Washington University, Washington, DC, United States; ^2^Department of Human and Molecular Genetics, Florida International University, Miami, FL, United States; ^3^Department of Research, The Focus Foundation, Davidsonville, MD, United States; ^4^Division of Genetics and Metabolism, Children's National Health System, Washington, DC, United States; ^5^Division of Neurogenetics and Developments Pediatrics, Children's National Health System, Washington, DC, United States; ^6^Department of Neurology, George Washington University, Washington, DC, United States

**Keywords:** Pallister-Killian syndrome, neurodevelopment, twin study, functional near-infrared spectroscopy, phenotype, genetics

## Abstract

Pallister–Killian syndrome is an uncommon genetic disorder that has broad developmental and multisystemic effects. While medical complications are widely reported throughout the literature, research on the neurodevelopmental profile has been limited. Case reports make up the majority of the few existing studies regarding the neurodevelopmental phenotype associated with this disorder. The current case report describes a 3-year-old male with Pallister–Killian syndrome (AF), reports the neurodevelopmental evaluation of his unaffected twin brother (MF), and outlines the results of an optical imaging study on both boys. AF presents with severe developmental delays, however, he ambulates with support and engages in conversation using his communication device. Most severely impaired was AF's speech and expressive language, with childhood apraxia of speech (CAS) as a possible explanation for these severe deficits. MF, the sibling, demonstrated neurotypical abilities and often advanced scores for his age. Both subjects completed a functional near-infrared spectroscopy (fNIRS) study, revealing decreased temporal and frontal lobe function in AF and typical functioning in MF. This case report expands on the existing literature on PKS by describing variances in fraternal twin presentation and novel reporting on fNIRS findings in both boys.

## Introduction

Pallister–Killian syndrome (PKS) is a rare, sporadic genetic disorder caused by mosaic tetrasomy of the short arm of chromosome 12 occurring in about 1 in 20,000 liveborn infants (1–6). Dysmorphic craniofacial features common to PKS include frontal bossing, alopecia, abnormal ears, hypertelorism, long philtrum, and sparse eyebrows or eyelashes (2–6). Additional characteristics consist of hypotonia, pigmentary skin and cardiac anomalies, hearing loss, visual impairment, gastrointestinal anomalies, and epilepsy (4–11).

The neurodevelopmental profile of PKS includes intellectual disability with severe delays in speech, language, and neuromotor skills (9, 12). Recently, more variability has been reported suggesting a milder phenotype in PKS (2, 9, 12–14). Blyth et al. (13) described 3 of 22 participants in their PKS cohort showing mild or moderate intellectual disability. Several children in this cohort were ambulatory, but only a handful of children had adequate speech abilities with more than a few words (13). Typical speech, motor, and cognitive development have also been outlined in case studies, further illustrating the variance in PKS (10, 12, 15, 16).

Speech and language dysfunction is largely characteristic of PKS, with first words typically occurring around 36 months (2, 4, 9, 12–14). Kostanecka et al. (9) reported that 14 of 16 participants were non-verbal with expressive language skills below 9 months of age. Similarly, Blyth et al. (13) reported that the majority of their cohort were non-verbal, where seven of the 22 participants (31.8%) used recognizable sounds, such as “mama” or “dada,” four (18.2%) had some sounds, and one used sign language (13).

Severe psychomotor delay is associated with PKS (2, 4, 9, 13). Izumi and Krantz described the average age for rolling as around 10.8 months, 21.2 months for sitting independently, and 38.8 months for walking (2). Kostanecka et al. (9) noted that 14 of 16 patients were non-ambulatory with fine motor skills largely delayed up to 7 months of age with severe hypotonia. Blyth et al. (13) reported that 40% of patients over 12 months could not sit without support. Nine children in this cohort were ambulatory and began walking between 16 months and 8 years (13).

Consistent with the neurodevelopmental findings, underlying brain abnormalities are common among patients with PKS (4, 7, 17). Neuroimaging studies revealed loss of cerebral brain volume, cortical malformations, corpus callosum dysgenesis, and abnormalities of the white matter (4, 7, 17). Barkovich (17) found perisylvian polymicrogyria, vermian dysplasia, brachium pontis signal deficits, meningeal anomalies, and unilateral assimilation of the atlas. These brain abnormalities often result in seizures, severe speech and language deficits, and motor delays (4, 7, 18). Perisylvian polymicrogyria, also known as congenital bilateral perisylvian syndrome (CBPS), has been associated with childhood apraxia of speech (CAS) and oral motor dysfunction, a common characteristic found in individuals with PKS (17).

Functional near-infrared spectroscopy (fNIRS) is a non-invasive optical imaging technology investigating the hemodynamics of the brain. It is an ideal modality when working with children with impaired motor function due to robustness of motion artifact (19, 20). It provides similar information to fMRI with more flexibility to investigate neurocognition in naturalistic settings outside the MRI scanner, allowing for more creative study designs (21, 22). The current case study describes the neurodevelopmental evaluation and fNIRS findings of a 3-year-old child with PKS and his fraternal twin brother.

## Patient Information

### Birth History

AF was the result of a twin pregnancy from a gravida-4, para-3, mis-2 35-year-old Caucasian female and her 39-year-old, non-consanguineous Caucasian partner. A fetal ultrasound at 20 weeks of gestation revealed the possibility of cystic hygroma and a narrow aortic isthmus in Twin A (AF). The non-invasive prenatal screening (NIPS) showed no chromosomal abnormalities, and an amniocentesis was declined. Maternal weight gain was approximately 29.48 kg. AF and his sibling (MF) were delivered at 36 weeks of gestation by cesarean section. AF weighed 3.13 kg (6.9 lbs, 25th percentile) and had APGAR scores of 1 and 8 at 1 and 5 min, respectively. MF weighed 2.10 kg (4.63 lbs, 1st percentile) with APGARs of 8 and 9 at 1 and 5 min, respectively. AF was treated for hyperbilirubinemia and neonatal hypoglycemia and was placed on oxygen and a cardiac monitor. All testing on MF was unremarkable.

### Perinatal History

Five days after birth, AF was transferred from a community hospital to a tertiary care hospital due to concerns about abdominal distention, the possibility of trisomy 21, and coarctation of the aorta. Dysmorphic features were evident: epicanthal folds, preauricular tag on the left ear, low-set ears, short philtrum, micrognathia, micropenis, undescended testes, and claw toes (Table 1). Due to these features, a karyotype and a chromosomal microarray were completed. Mosaic PKS was identified with an approximate 19 Mb gain of the terminal 12p from band p12.3. This gain resulted in 73% mosaicism for four copies of 12p in his DNA. AF remained hospitalized for 7.5 weeks and received both physical therapy and occupational therapy services during the hospitalization.

**Table 1:** Dysmorphic features of Pallister–Killian syndrome (PKS).

**Dysmorphic features of PKS**	
**Common features**	**Present in AF**
Epicanthal folds	✓
Preauricular tag	✓
Frontal bossing	✓
Low-set ears	✓
Long philtrum	✓
Alopecia	x
Micrognathia	✓
Sparse eyebrows/lashes	✓
Micropenis	✓
Claw toes	✓
Hypertelorism	x
Macroglossia	✓

*The checkmark indicates that AF presents with that specific abnormality*.

A 2-D echocardiogram revealed a closed patent ductus arteriosus (PDA), diminished left-sided cardiac structures with a small aorta, and aortic isthmus. However, his cardiac performance appeared adequate without any significant obstruction to the blood flow. At 2 weeks of age, he was evaluated by a pediatric otorhinolaryngologist for oxygen desaturations while in supine and during feeding, which improved with a change in position to side lying. Mild glossoptosis and slight arytenoid obstruction were identified. A pediatric neurologist noted truncal hypotonia with no pathologic reflexes and 2+ symmetric deep tendon reflexes. A brain MRI at 17 days of life revealed multiple intracranial hemorrhagic foci behind the cerebellum, hypothesized as a combination of subarachnoid and subdural blood products.

At approximately 1 month of age, AF was evaluated for obstructive sleep apnea with an apnea–hypopnea index of 58.6. He was placed on 2 kg of oxygen *via* nasal cannula. He had a direct laryngoscopy/bronchoscopy and a nasal trumpet placement, which found a grade II airway, macroglossia, and soft palate collapse. A gastrointestinal tube was placed due to feeding difficulties and a laparoscopic gastrostomy, which was removed at 11 months. At 14 months of age, an ABR revealed moderate to severe bilateral hearing loss. Bilateral cortical visual impairment was identified, with the lower right quadrant of the visual field believed to be the least impaired. Head drops were noted around 11 months of age, though currently, it remains unclear whether these are seizure related. EEG results identified occasional–frequent multifocal spike-wave discharges, sharp and slow wave discharges at T5, P3, O1, O2, T6, F3, and F4C4, and a slow posterior dominant rhythm. These findings indicate mild diffuse cerebral dysfunction and an increased risk of multifocal seizures, which is managed with a daily prescription of levetiracetam.

### Neurodevelopmental History

A review of the patient's developmental milestones, genetics, pediatric neurology, physical therapy, and occupational therapy reports were synthesized. At 7 months, he pivoted in prone, scooted on his stomach, and reached toward objects. He was not yet rolling but was beginning to prop on extended arms at 90° in a prone position. He would grasp objects and bring them to his mouth. He had crude transfer from hand-to-hand, and play at midline was emerging at this time.

At 18 months of age, AF received a gait trainer to facilitate independent ambulation and mobility. He developed transitions from prone in and out of sitting and to standing without support. He began using a modified parallel bar that assisted him into a standing position. By 24 months, AF was taking steps with support and using his gait trainer quite well. At 38 months, he began using body rolling as a form of independent movement and allowed for goal-directed mobility and attainment of objects across a long distance.

In expressive language, simple open vocalization sounds were produced at approximately 7 months. By 18 months, single consonant and vowel sounds were produced, but no multisyllabic or imitative vocalizations. By 26 months, he began saying “mama” and using some simple signs. He also received an Accent 1000 (AAC) device to address his hearing and vision losses. His communication was a combination of gestural language, some adapted signs, and his AAC device.

A review of AF's feeding history revealed a difficulty with latching from early infancy resulting in the placement of a gastrointestinal tube at 1 month of age and removal at 11 months. By 24 months, he could finger feed and had some spoon-feeding skills. AF is heavier than MF by approximately 9.07 kg, consistent with the overgrowth associated with PKS. The patient's diet is gluten-free due to a family history of celiac disease. He currently eats a wide variety of food and textures and drinks two to three 0.2-kg (7.05 oz) bottles of 2% milk per day.

## Materials and Methods

### Neurodevelopmental Assessments

The Wechsler Preschool and Primary Scales of Intelligence (WPPSI-IV), Expressive One-Word Picture Vocabulary Test (EOWPVT-4), and Receptive One-Word Picture Vocabulary Test (ROWPVT-4) were completed to assess MF's neurocognitive capabilities. Baseline scores on prior infant assessments could not be attained (i.e., Bayley). On the last visit, compliance improved enough to use age-appropriate neurodevelopmental assessments (WPPSI-IV, R/EOWPVT-4).

### Functional Near-Infrared Spectroscopy Paradigm

AF and MF participated in this study exploring brain activity while watching two 5-min-long videos with the functional near-infrared spectroscopy (fNIRS) cap on. The first was Peppa Pig (communicative language) followed by Timmy Time (non-communicative language without spoken dialog).

### Functional Near-Infrared Spectroscopy Data Acquisition

Subjects in this study were asked to sit in front of a monitor. An fNIRS sensor consisting of 16 sources and 16 detectors with a total of 16 source–detector distances (channels) of 3 cm (the cap) was placed on the subject's head. The fNIRS system was a NIRSport2 device from NIRx Medical Technologies, LLC (Berlin, Germany). It uses sources with two wavelengths of 760 and 850 nm to measure oxyhemoglobin and deoxyhemoglobin signals in cortical regions. The system uses the Aurora software to record raw light intensities.

### Functional Near-Infrared Spectroscopy Data Analysis

Signal processing was performed in the MATLAB R2019b environment using the raw intensities obtained from the NIRx machine. Total hemoglobin signal was evaluated for the comparison between AF and MF. Total hemoglobin is the summation of oxyhemoglobin and deoxyhemoglobin signals. Total signal was used in this study because it contained information from both signals. Preprocessing was completed on the collected signals to remove motion artifacts and the effects of the extracerebral layers. Based on our hypothesis, frontal, and temporal lobe channels were clustered and averaged for the analysis.

## Results

### Neurodevelopmental Evaluation—AF

The patient was referred by his clinical geneticist to our facility for a comprehensive neurodevelopmental evaluation at the age of 30 months. AF was attending school virtually because of COVID-19 with five other classmates: two typically developing and three with individualized education programs (IEP). He receives OT, PT, speech, and vision/mobility services through school as well as weekly private PT, and speech and language therapy. Additionally, he works with a separate communication device specialist targeting the implementation of his AAC, which he began at 2 years of age. AF has bilateral hearing aids, glasses, and an augmentative communication device to facilitate his communication. He was quite social, with gestural communication and body movements. He used his AAC, and his intent was typically clear.

He has decreased muscle tonus in the trunk and upper and lower extremities. His core strength remains diminished and is hampered by his overgrowth hindering him from initiating large motor movements. AF independently propels forward using a gait trainer and takes a few independent steps while holding onto rattan poles. He sits without support, frequently pivoting with ease and barrel rolling. He has protective extension responses in all directions. In fine motor, he shows competence with adaptive switches and pushes small buttons on his AAC with well-developed finger dissociation. He has supination and pronation of his forearm, which enables him to remove three pegs out of a pegboard among other activities.

AF is delayed in expressive language skills. However, he sequences multiple words using his AAC and utilizes gestural language to express his needs. For instance, parental report noted AF's ability to differentiate his various sounds, such as for hunger and fatigue. He follows single step related and unrelated directions. AF uses accurate eye gaze to match colors and objects in a book. He uses gestural body language to communicate by pushing objects or people away with his hands or turning himself away from undesired stimuli. A few vocalizations were heard but the mother reports that he is vocal with his siblings and is effective in communicating his wants and needs.

### Neurodevelopmental Evaluation—MF

MF presented as a typically developing child. On the WPPSI-IV, MF showed a full-scale intelligence quotient (FSIQ) of 110 at the 75th percentile. His composite scores were as follows (Table 2): Verbal Comprehension was 117, Visual Spatial was 97, Fluid Reasoning and Working Memory were 100, and Processing Speed was 94. On the EOWPVT-4 and the ROWPVT-4, he showed standard scores of 123 and 109, respectively (Table 2).

**Table 2:** MF neurodevelopmental evaluation.

**Wechsler preschool and primary scale of intelligence −4th edition**			
	**Sum of scaled scores**	**Composite** **score**	**Percentile** **rank**
Verbal comprehension	26	117	87
Visual spatial	19	97	42
Fluid reasoning	20	100	50
Working memory	20	100	50
Processing speed	18	94	34
Full-scale IQ	68	110	75
**Expressive and receptive one-word picture vocabulary tests–4th edition**			
	Raw score	Standard score	Percentile rank
EOWPVT-4	72	123	94
ROWPVT-4	62	109	73

### Functional Near-Infrared Spectroscopy Results

**Figure 1:**
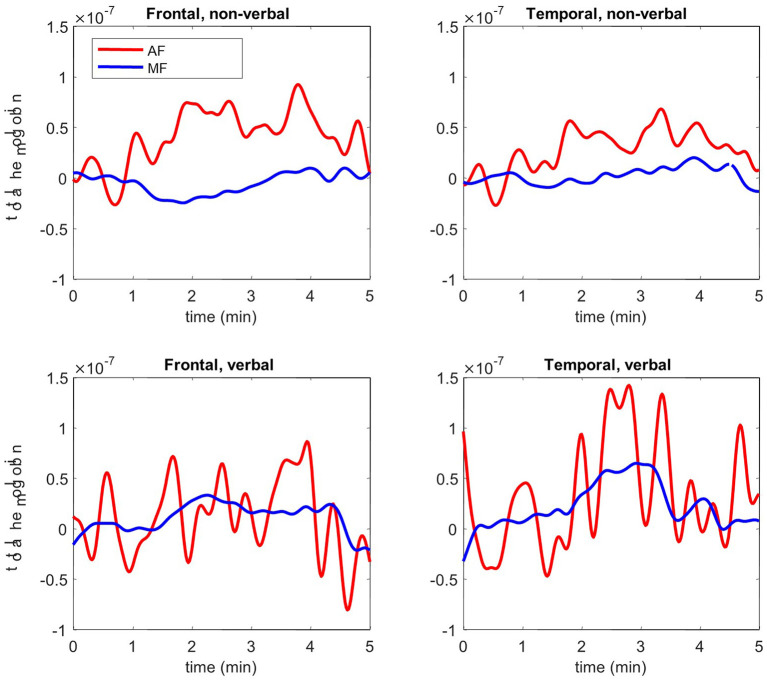
Functional near-infrared spectroscopy (fNIRS) results: total hemoglobin concentration of frontal and temporal lobes during 5 min of verbal and non-verbal tasks for AF (red) and MF (blue).

Total hemoglobin concentration signals show higher brain activation in temporal and frontal lobes during verbal and non-verbal tasks for AF (Figure 1). A significant difference was observed between the twins (*p* < 0.05) for the average amplitude of total hemoglobin over 5 min of both tasks (Table 3). These results confirm that temporal and frontal impairment is evident in AF, but not MF. Overall, the results of the fNIRS analysis hemodynamically confirm the impaired motor and cognitive processes on frontal and temporal lobes in AF. The higher brain activation corroborates the language and speech dysfunction, a hallmark manifestation of PKS.

**Table 3:** A comparison between hemodynamic activity of the frontal and temporal lobes during fNIRS.

**Hemodynamic activity of frontal and temporal lobes**				
**Total hemoglobin (μM)**		**AF**	**MF**	***P-*** **value**
Non-verbal	Frontal	0.04	0.01	0.02^*^
	Temporal	0.45	0.008	0.04^*^
Verbal	Frontal	0.063	0.012	0.007^*^
	Temporal	0.096	0.025	0.016^*^

*The*

* *indicates significance at the p < 0.05 level*.

## Discussion

The current case study describes the neurodevelopmental profile of a 3-year-old patient with PKS, a rare genetic disorder that affects all aspects of neurodevelopment. Though developmental delays were evident, AF presented with the ability to ambulate with support and respond to conversation through gestures and his AAC. He had age-appropriate receptive language skills and accurate eye gazing when asked to identify pictures in a book. These abilities suggest that AF, though significantly delayed in motor and expressive language, may be exhibiting more intact cognitive skills.

Case studies have demonstrated that the PKS phenotype is variable and may sometimes present with higher cognitive performance (12, 14). Bielanska et al. (12) presented a case study of a boy with “an atypical case of PKS with a mild phenotype.” He showed moderate gross motor and speech delay, but age-appropriate social and cognitive abilities at 3 years old (12). Stalker et al. (14) described a case of a girl diagnosed with PKS at 15 months of age. Her developmental testing indicated delays in neuromotor domains but typical neurocognitive abilities (14). Our case study and those previously described suggest that the intellectual abilities may be more variable than appreciated in PKS.

Consistent with AF's childhood apraxia of speech (CAS), motor planning difficulties are at the root of CAS and are often associated with neurogenetic disorders (23–25). These motor planning issues coupled with hypotonia often cause dysfunction with the orofacial musculature, resulting in difficulties shaping the muscles to articulate the desired production (24). AF's history of latching difficulties in infancy, oral motor hypotonia, speech delay, and articulation deficits suggest that CAS may be a fundamental component in his expressive language dysfunction. CAS rates among other neurodevelopmental disorders genetic in origin are reportedly increased, including the highest rate of 11.8% in 22q11.2 deletion syndrome (26). Little research has addressed the potential impact of CAS on expressive language dysfunction within PKS, and future studies should explore CAS as a possible contributing source for these deficits.

AF was the result of a twin pregnancy, which has scarcely been reported in the literature. Li et al. (27) reported on a twin pregnancy that was terminated when one twin was prenatally diagnosed with PKS. Additionally, Salzano (28) reviewed 142 cases of PKS where two cases were twin pregnancies with only one dizygotic and one monozygotic case. However, it was not reported whether these pregnancies resulted in live births. Therefore, this is the first report to our knowledge to document a live twin pregnancy resulting in one twin's diagnosis of PKS. Additionally, AF's twin was evaluated and presented neurotypical capabilities on all domains of development and met all developmental milestones at the appropriate times.

This is the first study, to our knowledge, to report the functional neuroimaging results of a patient with PKS and his twin. AF's results show significantly lower activation in the frontal and temporal lobes throughout both communicative and non-communicative tasks in comparison to MF. Poulton et al. (18) explored structural brain differences among patients with PKS and found that abnormalities of the perisylvian region, an area responsible for speech production, were frequently reported. Malformations of this area result in speech and language deficits that are often associated with CAS (29). Johnson et al. (30) investigated the use of fNIRS to explore brain functioning in the prefrontal cortex during treatment for CAS. Though fNIRS does not provide anatomical information, our results map cortical activation up to 1-cm deep during language-based assignments. AF's fNIRS results provide support for the neurodevelopmental findings and warrant exploration for CAS as an underlying rationale for expressive language dysfunction in PKS (18, 29).

AF presented with severe developmental delay and medical complications affecting all organ systems. This case study contributes to the wide variability of the PKS phenotype and hypothesizes CAS as the origin for the severe expressive language dysfunction common to PKS. There is a paucity of literature addressing the core etiology of deficient verbal abilities in PKS, and additional research is warranted regarding the potential presence of CAS in association with PKS. This is the first study to investigate brain differences among a pediatric patient with PKS and his neurotypical twin. With advances in neuroimaging, we may be able to understand the deficient neuronal underpinnings of this disorder and help guide clinicians in more targeted services.

## Data Availability Statement

The datasets for this article are not publicly available due to concerns regarding participant/patient anonymity. Requests to access the datasets should be directed to the corresponding author.

## Ethics Statement

The studies involving human participants were reviewed and approved by Western IRB. Written informed consent to participate in this study was provided by the participants' legal guardian/next of kin. Written informed consent was obtained from the individual(s), and minor(s)' legal guardian/next of kin, for the publication of any potentially identifiable images or data included in this article.

## Author Contributions

MH, CS-S, and AG: conceptualization. KR, TS, and ET: data curation. CS-S, KR, KK, FM, ET, and AG: clinical management. KK: formal analysis. CS-S, KR, FM, and AG: resources. CS-S, KR, FM, ET, and AG: supervision. CS-S, MH, and ET: writing—original draft. MH, CS-S, KR, RK, MB, ET, and AG: writing—review and editing. All authors contributed to the article and approved the submitted version.

## Conflict of Interest

The authors declare that the research was conducted in the absence of any commercial or financial relationships that could be construed as a potential conflict of interest.

## Publisher's Note

All claims expressed in this article are solely those of the authors and do not necessarily represent those of their affiliated organizations, or those of the publisher, the editors and the reviewers. Any product that may be evaluated in this article, or claim that may be made by its manufacturer, is not guaranteed or endorsed by the publisher.
