# Supplementary figures and images for: Case Report: A Case Study on the Neurodevelopmental Profile of a Child With Pallister–Killian Syndrome and His Unaffected Twin
Source: Front Pediatr. 2022 Mar 15;10:817133. doi: 10.3389/fped.2022.817133 (PMC8965074; doi:10.3389/fped.2022.817133)

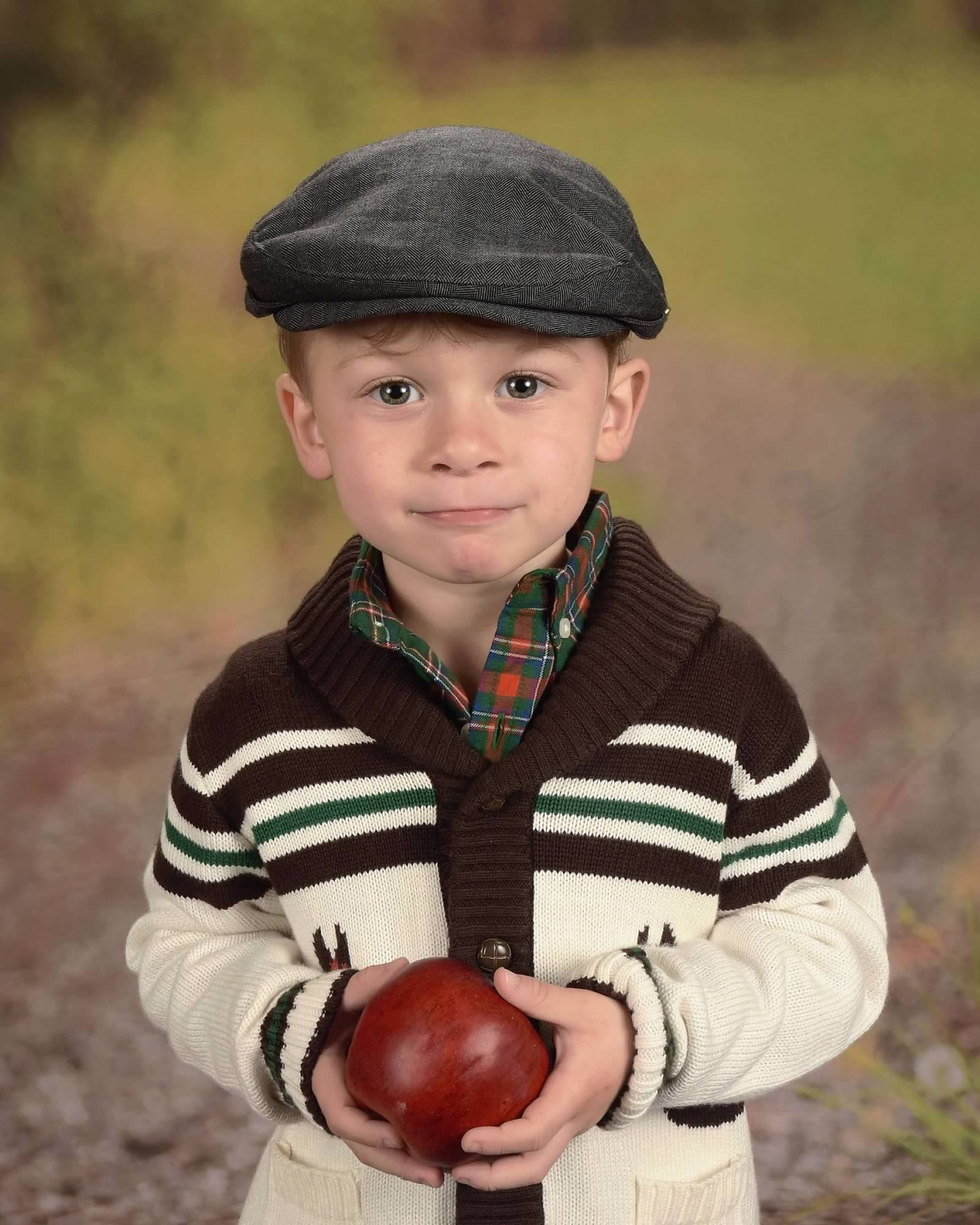

Supplement: Supplementary file 2 [file Image_2.JPEG]
